# Chest compression-related fatal internal mammary artery injuries manifesting after venoarterial extracorporeal membrane oxygenation: a case series 

**DOI:** 10.1186/s13256-017-1485-y

**Published:** 2017-11-11

**Authors:** Toshinobu Yamagishi, Masahiro Kashiura, Kazuhiro Sugiyama, Kazuha Nakamura, Takuto Ishida, Takahiro Yukawa, Kazuki Miyazaki, Takahiro Tanabe, Yuichi Hamabe

**Affiliations:** 1 Tertiary Emergency Medical Center, Tokyo Metropolitan Bokutoh Hospital, 4-23-15 Kotobashi, Sumida-ku, Tokyo, 130-8575 Japan; 2 Department of Emergency and Critical Care Medicine, Jichi Medical University Saitama Medical Center, 1-847 Amanuma-cho, Omiya-ku, Saitama-shi, Saitama 330-8503 Japan

**Keywords:** Extracorporeal membrane oxygenation, Cardiopulmonary resuscitation, Mammary arteries, Hemorrhagic shock, Therapeutic embolization, Heparin, Platelet aggregation inhibitors, Induced hypothermia

## Abstract

**Background:**

Cardiopulmonary resuscitation-related bleeding, especially internal mammary artery injuries, can become life-threatening complications after initiating venoarterial extracorporeal membrane oxygenation owing to the frequent involvement of concomitant anticoagulant treatment, antiplatelet treatment, targeted temperature management, and bleeding coagulopathy. We report the cases of five patients who experienced this complication and discuss their management.

**Case presentation:**

We retrospectively evaluated five patients with cardiopulmonary resuscitation-related internal mammary artery injuries who were treated between February 2011 and February 2016 at our institution. All five patients were Asian men, aged 56 to 68-years old, who had received concomitant intravenously administered unfractionated heparin (3000 units) with antiplatelet therapy. Four patients received targeted temperature management. The injuries and hematomas were detected using contrast-enhanced computed tomography in all cases. Three patients were treated using transcatheter arterial embolization within 6 hours following cardiopulmonary arrest, and two were resuscitated and received appropriate treatment following early recognition of their injuries. Two patients died of hemorrhagic shock with delayed intervention. Four of the five patients had excessively prolonged activated partial thromboplastin times before their interventions.

**Conclusions:**

Computed tomography should be performed as soon as possible after the return of spontaneous circulation to identify injuries and consider appropriate treatments for patients who have experienced cardiac arrest. Delayed bleeding may develop after treating hypovolemic shock and relieving arterial spasms; therefore, transcatheter arterial embolization should be performed aggressively to prevent delayed bleeding even in the absence of extravasation. This approach may be superior to thoracotomy because it is less invasive, causes less bleeding, and can selectively stop arterial bleeding sooner. A 3000-unit intravenous bolus of unfractionated heparin may be redundant; heparin-free extracorporeal cardiopulmonary resuscitation may be a more appropriate alternative. Unfractionated heparin treatment can commence after the bleeding has stopped.

## Background

Chest compressions for cardiopulmonary resuscitation (CPR) were emphasized in the 2010 American Heart Association CPR guidelines [1]. However, CPR-related bleeding complications are not uncommon [2–4], and CPR-related fractures have been observed more frequently since 2010 [5, 6]. Furthermore, CPR-related bleeding, particularly internal mammary artery (IMA) injuries, can become life-threatening in patients who undergo extracorporeal cardiopulmonary resuscitation (ECPR) because concomitant anticoagulant treatment [7], antiplatelet treatment, targeted temperature management [8], and bleeding coagulopathy [9] are often involved. We investigated five patients with CPR-related IMA injuries that occurred after they had undergone venoarterial extracorporeal membrane oxygenation (VA-ECMO).

## Case presentations

We retrospectively evaluated the medical records of patients with cardiac arrest who underwent VA-ECMO between February 2011 and February 2016 at our institution. Of the 129 patients identified, five experienced CPR-related IMA injuries. Four of these patients underwent ECPR and one underwent VA-ECMO for cardiogenic shock after the return of spontaneous circulation (ROSC). All five patients were Asian men who had received 3000 units of intravenously administered unfractionated heparin with concomitant antiplatelet therapy for suspected acute coronary syndrome. Four of the five patients underwent targeted temperature management (the exception being Patient 3). Contrast-enhanced computed tomography (CT) revealed CPR-related chest bleeding complications in all cases, for example, IMA injuries, intercostal artery (ICA) injuries, mediastinum hematoma, and hemothorax.

### Case 1

**Fig. 1:**
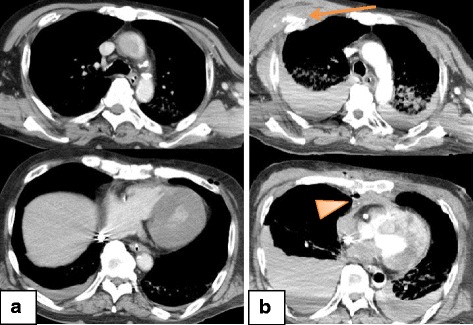
Computed tomography findings from Patient 1. **a** Contrast-enhanced computed tomography after the extracorporeal cardiopulmonary resuscitation revealed bilateral multiple rib fractures, a sternum fracture, and right pleural effusion without contrast extravasation. **b** Repeat contrast-enhanced computed tomography after coronary angiography revealed contrast extravasation at the anterior chest wall (*arrow*) and mediastinum (*arrowhead*), with increases in the size of these hematomas

A 65-year-old Asian man had a history of myocardial infarction and atrial fibrillation, and was receiving dual antiplatelet and warfarin treatment. After collapsing at a concert, he received immediate bystander CPR as well as six automated external defibrillator shocks before the emergency medical service (EMS) arrived; he was then transported to our hospital. His rhythm was asystole at the time of admission, and VA-ECMO commenced 45 minutes after his cardiac arrest because conventional CPR did not result in ROSC. Contrast-enhanced CT after starting VA-ECMO revealed bilateral multiple rib fractures, a sternum fracture, and right pleural effusion without contrast extravasation (Fig. 1a). Transcatheter arterial embolization (TAE) was not indicated; coronary angiography (CAG) was performed for suspected cardiogenic cardiac arrest based on the prehospital shockable rhythm. His left circumflex artery was completely occluded, and percutaneous coronary intervention (PCI) was attempted but failed because of strong calcification. Repeat contrast-enhanced CT was performed because of unstable blood flow during the VA-ECMO, and our patient exhibited a gradual decrease in his hemoglobin (Hb) levels from 10.8 g/dL at admission to 8.4 g/dL 2 hours later. CT revealed contrast extravasation at his mediastinum and right anterior chest wall, with increases in the sizes of the hematomas (Fig. 1b). TAE was performed for his right IMA and right ICA using n-butyl cyanoacrylate (NBCA) 560 minutes after his cardiac arrest. His activated partial thromboplastin time was > 130 seconds before TAE. He subsequently died 2 hours after TAE despite receiving 26 red blood cell units, 16 fresh frozen plasma units, and three packs of cryoprecipitate.

### Case 2

**Fig. 2:**
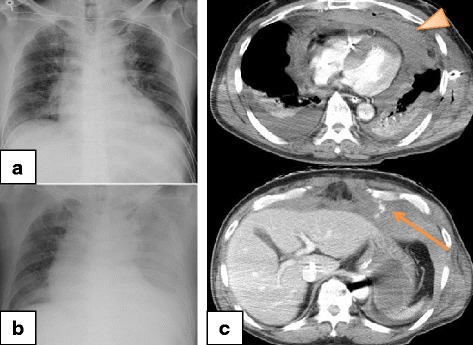
Chest radiography and computed tomography findings from Patient 2. **a** Chest radiography at the time of admission did not reveal any traumatic abnormalities. **b** Chest radiography 3 days after the admission revealed decreased left lung permeability. **c** Contrast-enhanced computed tomography from after the insertion of a chest drainage tube revealed extravasation of the contrast agent behind the left sixth rib (*arrow*), a hematoma at the mediastinum (*arrowhead*), and chest cavities without any obvious rib fractures

A 68-year-old Asian man was transported to another hospital by the EMS because of chest pain. Ventricular fibrillation (VF) was noted on his arrival, and conventional CPR was performed immediately. He achieved ROSC after four defibrillation shocks, and was subsequently transferred to our hospital for post-cardiac arrest care. An intra-aortic balloon pump (IABP) was inserted at our hospital for cardiogenic shock, and CAG was also performed, revealing total occlusion of his left anterior descending artery. He was diagnosed as having acute myocardial infarction and underwent successful PCI. Chest radiography did not reveal any traumatic abnormalities (Fig. 2a), and his hemodynamics were maintained using IABP and a continuous infusion of noradrenalin. Minimal change was observed in his Hb levels (admission, 13.5 g/dL; 6 hours later, 13.3 g/dL). Two days after admission, he suddenly deteriorated, and VA-ECMO was initiated for suspected cardiogenic shock. However, his Hb level decreased to 5.3 g/dL, which indicated hemorrhagic shock. Ultrasonography and chest radiography revealed massive left pleural effusion (Fig. 2b), and a massive hemothorax was diagnosed after inserting a chest drainage tube. The blood flow from the drainage tube was > 200 mL/hour for several hours, and contrast-enhanced CT was performed to identify the source of the bleeding. Contrast extravasation was noted behind his left sixth rib, as well as hematoma at his mediastinum and hemothorax without rib fracture (Fig. 2c). Thoracotomy was performed to achieve hemostasis 48 hours after cardiac arrest. Although the bleeding appeared to originate from his left ICA, it was difficult to stop; therefore, an artery involved with the adjacent rib was also ligated. However, this procedure did not stop the bleeding from the left chest drain, and angiography at 8 hours after the thoracotomy revealed bleeding from his left IMA. TAE was performed using microcoils and gelatin sponge pledgets, but did not control the bleeding. He subsequently died because of hemorrhagic shock 8 hours after the TAE.

### Case 3

**Fig. 3:**
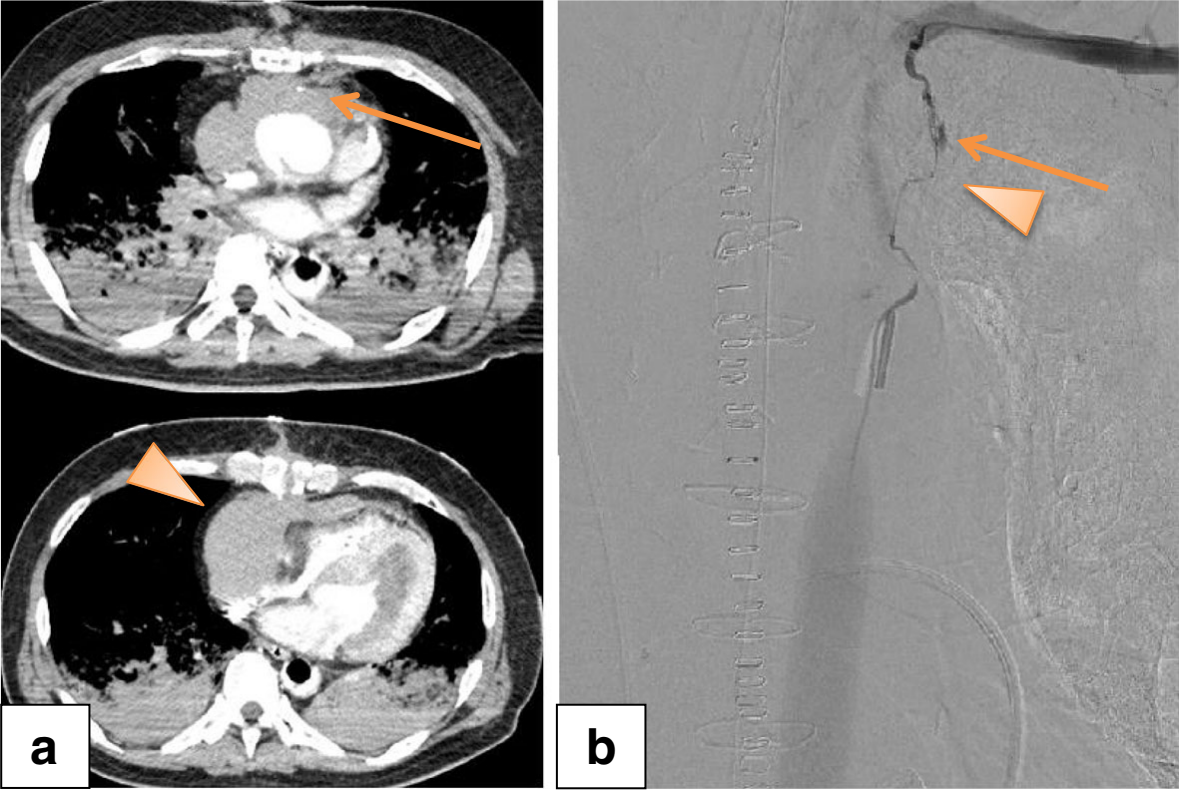
Computed tomography and angiography findings from Patient 3. **a** Contrast-enhanced computed tomography after the percutaneous coronary intervention revealed contrast extravasation (*arrow*), a hematoma at the mediastinum (*arrowhead*), and compression of the right atrium and ventricle. **b** Angiography after the contrast-enhanced computed tomography revealed contrast extravasation proximal to the left internal mammary artery (*arrow*) with arterial spasm (*arrowhead*)

A 56-year-old Asian man (an in-patient) had undergone an emergency graft implantation for a type A aortic dissection. On the eighth day post-surgery, he lost consciousness and experienced cardiac arrest that was witnessed by a surgeon in the general ward. He experienced refractory VF after conventional CPR, and VA-ECMO was started 28 minutes after the cardiac arrest. CAG revealed a thrombus between the aortic graft and the left main trunk of his coronary artery. Blood flow in his coronary artery improved after thrombus aspiration and balloon angiography. Contrast-enhanced CT after the PCI revealed extravasation and a hematoma at the mediastinum (Fig. 3a). Angiography revealed that the extravasation was proximal to his left IMA (Fig. 3b). Although TAE was performed using NBCA at 313 minutes after his cardiac arrest, the spasm persisted and ECMO flow could not be maintained at that time. He died 41 minutes after the TAE.

### Case 4

**Fig. 4:**
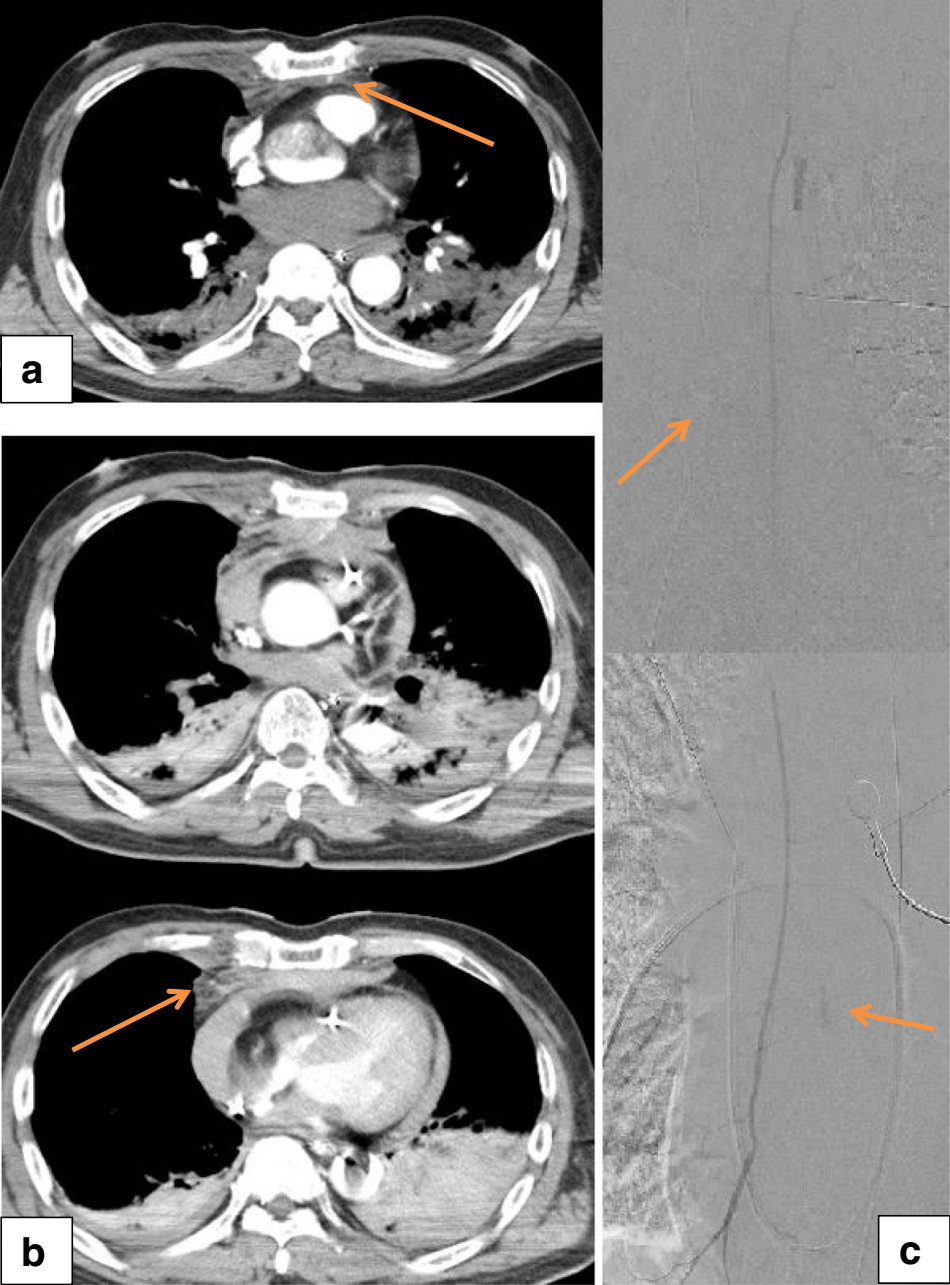
Computed tomography and angiography findings from Patient 4. **a** Contrast-enhanced computed tomography before the coronary angiography revealed a sternal fracture and a retrosternal hematoma with contrast extravasation of contrast (*arrow*). **b** Repeat computed tomography after the percutaneous coronary intervention revealed new contrast extravasation behind the fifth costal cartilage and beside the sternum (*arrow*), as well as growing hematomas in the retrosternal space. **c** Angiography after the percutaneous coronary intervention revealed bilateral contrast extravasation at the bilateral internal mammary arteries (*arrow*s)

A 62-year-old Asian man was transported to our hospital by the EMS because of pulseless electrical activity after receiving bystander CPR. VF was documented at the admission, and VA-ECMO was started for refractory VF 55 minutes after his cardiac arrest. Contrast-enhanced CT revealed a sternal fracture and a retrosternal hematoma with contrast extravasation (Fig. 4a). CAG was initially performed because the VA-ECMO flow was maintained and revealed 90% stenosis of his left main trunk. He was diagnosed as having acute myocardial infarction and underwent successful PCI. IABP was initiated to treat cardiogenic shock during the VA-ECMO. He exhibited a gradual decrease in his Hb levels: admission, 16.3 g/dL; 4 hours later, 14.2 g/dL. Repeat contrast-enhanced CT was performed because of the hematoma and contrast extravasation, and revealed new contrast extravasation behind his right fifth costal cartilage and beside his sternum, as well as growing hematomas in the retrosternal space (Fig. 4b). Angiography revealed extravasations at his bilateral IMAs, and TAE was performed using NBCA at 327 minutes after his cardiac arrest (Fig. 4c). The TAE successfully decreased the IMA flow and the extravasations disappeared. The VA-ECMO and IABP were tapered on day 7 and day 11 post-admission, respectively. He was subsequently transferred to another hospital for rehabilitation on day 123 at which point his Glasgow–Pittsburgh cerebral performance category was 3.

### Case 5

**Fig. 5:**
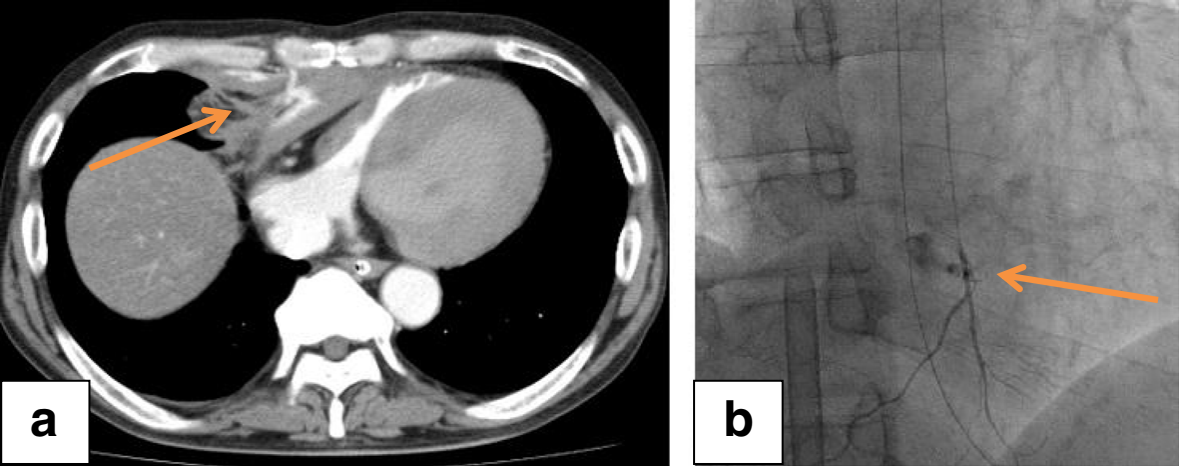
Computed tomography and angiography findings from Patient 5. **a** Contrast-enhanced computed tomography from before the coronary angiography revealed right rib fractures, a sternum fracture, and a hematoma at the mediastinum with contrast extravasation (*arrow*). **b** Angiography after the coronary angiography revealed contrast extravasation at the right internal mammary artery (*arrow*)

A 62-year-old Asian man was transported to our hospital by the EMS because of VF after four defibrillation shocks. VA-ECMO was started for refractory VF at 40 minutes after his cardiac arrest. Contrast-enhanced CT before the CAG revealed right rib fractures, a sternum fracture, and a mediastinal hematoma with contrast extravasation (Fig. 5a). CAG did not reveal any coronary artery stenosis, and selective right IMA angiography subsequently identified the extravasation (Fig. 5b). Artery embolization was performed using gelatin sponge pledgets and microcoils at 181 minutes after his cardiac arrest, and IABP was started after the TAE. He was a heavy drinker and was diagnosed as having VF that was caused by hypomagnesemia (serum magnesium level of 1.7 mg/dL at admission) and hypokalemia (serum potassium level of 2.6 mmol/L at admission). The VA-ECMO and IABP were tapered on days 2 and 4 post-admission, respectively. After implantation of a cardiac defibrillator, he was transferred to another hospital for rehabilitation on day 88, when he exhibited a cerebral performance category of 3.

## Discussion

We retrospectively identified five cases of CPR-related IMA injuries that were detected after VA-ECMO had been initiated (Tables 1, 2, and 3). These cases highlighted three important clinical principles. First, patients who have been resuscitated after cardiac arrest should undergo CT as soon as possible to identify any CPR-related injuries (especially IMA injuries), which can also help select the appropriate treatment(s). Second, TAE may be superior to thoracotomy for treating IMA injuries. Third, an initial 3000-unit intravenous bolus of heparin may be redundant in cases that involve ECPR.

**Table 1:** Patient characteristics

Case	Age (years)	Sex	Initial rhythm	ECPR	Time from CPR to VA-ECMO (minutes)	Diagnosis	Past medical history	Oral medicine
1	65	Male	VF?	Yes	45	AMI	AF, AMI, DM, HT, HL	Aspirin, clopidogrel, warfarin
2	68	Male	VF	No	1857	AMI	HT	N.p.
3	56	Male	VF	Yes	28	AMI	HT, type A aortic dissection	Aspirin
4	62	Male	PEA	Yes	55	AMI	N.p.	N.p.
5	62	Male	VF	Yes	40	Electrolyte abnormality	N.p.	N.p.

*AF* atrial fibrillation, *AMI* acute myocardial infarction, *CPR* cardiopulmonary resuscitation, *DM* diabetes mellitus, *ECPR* extracorporeal cardiopulmonary resuscitation, *HL* hyperlipidemia, *HT* hypertension, *N.p.* nothing particular, *PEA* pulseless electrical activity, *VA-ECMO* venoarterial extracorporeal membrane oxygenation, *VF* ventricular fibrillation

**Table 2:** Patient clinical outcome

Case	Extravasation in first CT	Location of bleeding	Intervention	Intervention time from cardiac arrest (minutes)	Death from intervention (minutes)	Survival to discharge
1	No	Rt. ICA/Rt. IMA	TAE	560	143	Died
2	Yes	Lt. IMA	Operation → TAE	2908	367	Died
3	Yes	Lt. IMA	TAE	313	41	Died
4	Yes	Bi. IMA	TAE	327	NA	CPC3
5	Yes	Rt. IMA	TAE	181	NA	CPC3

*Bi.* bilateral, *CPC* cerebral performance category, *CT* computed tomography, *ICA* intercostal artery, *IMA* internal mammary artery, *Lt.* left, *NA* not applicable, *Rt.* right, *TAE* transcatheter arterial embolization

**Table 3:** Blood test before intervention started and transfusion until intervention finished

Case	Hb (g/dL)	Plt (104/μL)	APTT (seconds)	Fibrinogen (mg/dL)	RBC (U)	FFP (U)	PC (U)	Cryoprecipitate (pack)
1	8.4	18.5	>130	126	26	16	0	3
2	5.3	7.1	>130	170	16	10	20	0
3	6.7	8.4	>130	124	12	10	10	0
4	14.2	14.1	49.7	169	0	6	0	0
5	10.1	7	>130	115	0	0	0	0

*APTT* activated partial thromboplastin time, *FFP* fresh frozen plasma, *Hb* hemoglobin, *PC* platelet concentrate, *Plt* platelet, *RBC* red blood cell, *U* Unit

A retrospective study of CT-detected CPR-related injuries revealed that 10% of cases involved chest wall hematomas, while 15% involved secondary signs of sternal fracture (retrosternal or mediastinal hematomas) [2]. In addition, a prospective study revealed that the incidence of CT-detected hemothorax was 11.3% [3]. Thus, CPR-related bleeding is not a rare complication, although the bleeding sources and their frequencies remain unclear. For example, bleeding can be caused by venous hemorrhage due to rib or sternal fracture [4] and/or arterial and branch hemorrhage due to ICA or IMA injuries. Although the 2015 CPR guidelines recommend targeting a reduced minimum compression depth (5 cm) [10], chest injuries have not been eliminated. On the other hand, the 2015 American Heart Association CPR guidelines recommend considering ECPR during a limited period of mechanical cardiorespiratory support for select patients with cardiac arrest and a suspected reversible etiology [11], as this approach may improve neurological outcomes [12]. While there are not many reports of CPR-related chest bleeding complications after ECPR [9], life-threatening chest bleeding complications may become increasingly common because of the new guideline recommendations.

IMA injuries can result in potentially life-threatening hemorrhage within a few minutes because of the rapid blood flow (150 mL/minute) [13, 14]. However, the patient described in Case 2 exemplifies the fact that chest radiography may not reveal indirect signs of IMA injuries (for example, slight hemothorax or a mediastinal hematoma) [13, 15], and early CT is required to identify active IMA bleeding and guide rapid surgical or angiographic intervention [15, 16]. Cases 3 to 5 involved TAE treatment within 6 hours after the cardiac arrest, and the successful resuscitations and treatments in Cases 4 and 5 were based on the early CT findings. Unfortunately, patient 3 died because of hemorrhagic shock, as early intervention was unsuccessful because of arterial spasm. Of interest, IMA bleeding can result in temporary hemostasis because of arterial spasm and hypotension, which can lead to delayed bleeding after these complications are treated [13, 14, 17]. The deaths of patients 1 and 2 were caused by hemorrhage shock because of the delayed treatment. The TAE in patient 1 was performed > 9 hours after the cardiac arrest because contrast extravasation was not detected during the first CT. In patient 2, the thoracotomy was performed at > 48 hours after the cardiac arrest because he did not undergo CT at his admission and exhibited stable Hb levels during a 6-hour period. Therefore, if the initial CT reveals massive hemorrhage, angiography should be considered to temporarily achieve hemostasis and delay bleeding. Furthermore, TAE may be performed aggressively in cases with IMA injuries, even if no active extravasation is detected during angiography [17].

Early contrast-enhanced CT should be performed to identify any CPR-related injuries and their etiologies, which can determine the indication for early TAE or thoracotomy. The selection of TAE or thoracotomy remains controversial, although surgery may be the first choice if the patient’s condition does not permit angiography [13]. Furthermore “damage control interventional radiography” uses a time-sensitive algorithm that prioritizes saving the life of a hemorrhaging patient, and may be performed for hemodynamically unstable patients after a traumatic injury [18]. A previous study revealed success rates of 91.6% for the patients in an embolization group and 66% for those in a surgically managed group [14]. The death of patient 2 may have been caused by surgical invasion and bleedings associated with the procedures combined with the concomitant intravenously administered anticoagulant treatment (unfractionated heparin) under VA-ECMO. Thus, TAE may be superior to thoracotomy because it is less invasive, causes less bleeding, and selectively stops arterial bleeding earlier than surgery (for example, in patients 4 and 5). Hence, we recommend that TAE be considered before surgery in cases that involve VA-ECMO, although exceptions may be made for patients in whom a massive hematoma compresses the right atrium or superior vena cava (such as in patient 3). In these circumstances, surgical treatment may be required to remove the hematoma and facilitate VA-ECMO blood flow.

The activated partial thromboplastin time before the surgery or TAE was excessively prolonged in all cases except for patient 4. Thus, if CT reveals CPR-related injuries but no interventions are planned, delayed bleeding may develop in association with arterial spasm and hypotension. In such cases, it may be useful to normalize the patient’s coagulation status and perform careful systemic heparinization after starting VA-ECMO. The guidelines for extracorporeal life support recommend a heparin bolus (50 to 100 units/kg) at the time of cannulation [7], although this recommendation is not specific to ECPR. Furthermore, a retrospective study of VA-ECMO in patients with severe trauma with hemorrhagic shock revealed that the mean duration of heparin-free ECMO was 5 days, and that the patients did not experience thromboembolic events or unexpected blood clot formation [19]. Therefore, initially heparin-free ECPR may be optimal for reducing bleeding complications, and heparin could be administered after confirming that hemostasis has been achieved using angiography or repeat CT [20].

## Conclusions

Patients who are resuscitated after cardiac arrest should undergo contrast-enhanced CT as soon as possible to identify any CPR-related injuries, especially IMA injuries, and to determine the appropriate course of treatment. TAE may be superior to thoracotomy for treating IMA injuries, although a 3000-unit intravenous bolus of heparin may be redundant at the start of ECPR.

## Abbreviations

CAG: Coronary angiography
CPR: Cardiopulmonary resuscitation
CT: Computed tomography
ECMO: Extracorporeal membrane oxygenation
ECPR: Extracorporeal cardiopulmonary resuscitation
EMS: Emergency medical service
Hb: Hemoglobin
IABP: Intra-aortic balloon pump
ICA: Intercostal artery
IMA: Internal mammary artery
NBCA: N-butyl cyanoacrylate
PCI: Percutaneous coronary intervention
ROSC: Return of spontaneous circulation
TAE: Transcatheter arterial embolization
VA-ECMO: Venoarterial extracorporeal membrane oxygenation
VF: Ventricular fibrillation
